# Short-term meditation increases blood flow in anterior cingulate cortex and insula

**DOI:** 10.3389/fpsyg.2015.00212

**Published:** 2015-02-26

**Authors:** Yi-Yuan Tang, Qilin Lu, Hongbo Feng, Rongxiang Tang, Michael I. Posner

**Affiliations:** ^1^Department of Psychological Sciences, Texas Tech UniversityLubbock, TX, USA; ^2^Department of Psychology, University of OregonEugene, OR, USA; ^3^Institute of Neuroinformatics and Lab for Body and Mind, Dalian University of TechnologyDalian, China; ^4^First Affiliated Hospital of Dalian Medical UniversityDalian, China; ^5^Department of Psychology, The University of Texas at AustinAustin, TX, USA

**Keywords:** integrative body–mind training, cerebral blood flow, positive mood, frontal asymmetry, anterior cingulate cortex

## Abstract

Asymmetry in frontal electrical activity has been reported to be associated with positive mood. One form of mindfulness meditation, integrative body-mind training (IBMT) improves positive mood and neuroplasticity. The purpose of this study is to determine whether short-term IBMT improves mood and induces frontal asymmetry. This study showed that 5-days (30-min per day) IBMT significantly enhanced cerebral blood flow (CBF) in subgenual/adjacent ventral anterior cingulate cortex (ACC), medial prefrontal cortex and insula. The results showed that both IBMT and relaxation training increased left laterality of CBF, but only IBMT improved CBF in left ACC and insula, critical brain areas in self-regulation.

## INTRODUCTION

Mindfulness meditation has been shown to produce positive effects on psychological wellbeing (Hölzel et al., 2011). As an important benefit of meditation practice, changes in self-reported positive mood or emotion are often observed (Kabat-Zinn et al., 1992; Tang et al., 2007). In particular, one form of meditation, the integrative body-mind training (IBMT) that originates from traditional Chinese medicine, improves attention, self-regulation, and mood after only few hours of training in comparison with relaxation training in a random assignment design (Tang et al., 2007; Ding et al., 2014).

Since late 1970s, frontal EEG asymmetry has had widespread use in measuring individual differences in emotional state. In general, it is believed a left-sided frontal activation indicates a positive emotion, although the evidence is not always consistent with this view (Allen and Kline, 2004; Allen et al., 2004; Cacioppo, 2004; Travis and Arenander, 2004, 2006).

A number of EEG studies have examined the relationship between meditation and frontal asymmetry (Davidson et al., 2003; Moyer et al., 2011). For example, 8-weeks mindfulness-based stress reduction, in comparison to a wait-list control, increased left-sided lateralization of alpha power and decreased negative affect (Davidson et al., 2003). Another study suggested 5-weeks of meditation shifts EEG asymmetry toward a pattern associated with positive emotion compared to a waiting-list control although there was no significant difference between the two groups (Moyer et al., 2011).

However, previous studies have used EEG with low spatial resolution and have not involved an active control group. To our knowledge, only one single photon emission computed tomography (SPECT) imaging study reported a marginal significant left frontal asymmetry of cerebral blood flow (CBF) but this study involved a sample of 12 long-term meditators compared to normal controls (Newberg et al., 2010) and was not a randomized test with an active control. Thus, we set out to apply brain imaging to investigate the CBF asymmetry induced by short-term training in a relatively large sample of 40 undergraduates with a random assignment to IBMT or relaxation training groups. We hypothesize that IBMT could improve left frontal CBF at resting state, which may underlie the promotion of positive emotion.

## MATERIALS AND METHODS

### PARTICIPANTS

Forty right-handed Chinese undergraduates at Dalian University of Technology without any training experience and a history of psychiatric or neurological conditions were recruited. They were randomly assigned to IBMT or relaxation group (20:20 each group, age = 22.75 ± 2.02), a brief self-report mood scale, similar to short form of Positive and Negative Affect Schedule (PANAS) was used to measure positive (PA) and negative affect (NA; Moyer et al., 2011; Ding et al., 2014). A written informed consent was obtained and local IRB approved the study.

### TRAINING

Participants received 30-min of IBMT or relaxation training from Monday through Friday, with a total of 2.5 h training. IBMT involves body relaxation, mental imagery, and mindfulness training, guided by an IBMT coach and compact disk. Cooperation between the body and the mind is emphasized in facilitating and achieving a meditative state. The trainees concentrated on achieving a balanced state of body and mind. The method stresses no effort to control thoughts, but instead a state of restful alertness that allows a high degree of awareness of body, mind, and external instructions (Tang et al., 2007, 2010; Tang, 2011). Relaxation training involves the relaxing of different muscle groups over the face, head, shoulders, arms, legs, chest, back, and abdomen, etc., guided by a tutor and compact disk. With eyes closed and in a sequential pattern, one is forced to concentrate on the sensation of relaxation such as the feelings of warmth and heaviness. This progressive training helps the participant achieve physical and mental relaxation and calmness (Tang et al., 2007, 2010).

### IMAGE ACQUISITION AND ANALYSIS

Single photon emission computed tomography neuroimaging data were acquired on a double head detector of GE HAWK EYE SPECT system (Millenium VG, GE Healthcare) from all participants before and after a 5-days IBMT or relaxation training. Participants were instructed to stay in a quiet dark room with eyes closed and ears unoccluded for 10 min, and then they were injected with 25 mCi of ^99m^Tc-ECD. Approximately 20 min following the injection, participants were scanned by SPECT system for 30 min (Tang et al., 2009; Newberg et al., 2010). Two different statistical methods were used to estimate CBF before and after training.

Statistical parametric mapping (SPM ^1^) was applied to evaluate whole brain CBF differences between these two groups. All images were normalized to the SPECT template in SPM, and then 8 mm Gaussian kernel was used to smooth these images. Two groups’ data were compared using two sample *t*-test with *ANCOVA* for removing global effect. The significance threshold was set at *p* < 0.001 (Tang et al., 2009; Newberg et al., 2010). The location and peaks of activation was identified by xjview ^2^.

Frontal asymmetry analysis was performed by SPSS (SPSS Inc., Chicago, IL, USA). CBF of the prefrontal regions, including inferior, middle, and superior frontal gyrus were extracted. All brain regions were identified by AAL atlas (Tzourio-Mazoyer et al., 2002). The laterality indexes were calculated by the following formula (Newberg et al., 2010):

$$Laterality\,\;Index=\;\frac{\left(Right-Left\right)}{0.5\,\;*\;\left(Right+Left\right)}*100$$

## RESULTS

Consistent with our previous studies (Tang et al., 2007; Ding et al., 2014), no significant difference in mood was detected before training between two groups (*p* > 0.05). After training, the IBMT group showed significantly better scores in positive and negative affect in comparison with the RT group; IBMT group (not RT) also showed significant post vs. pre difference in PN and NA (all *p* < 0.05). These results suggested that short-term IBMT induced higher positive mood and lower negative mood states than relaxation training. However there was no significant time by group interaction.

Imaging results showed that two groups did not differ in frontal CBF asymmetry before training (*p* > 0.05). However, the IBMT group (not relaxation group) had a significant CBF increase in subgenual/adjacent ventral anterior cingulate cortex (ACC; BA 25, BA 32), medial prefrontal cortex (BA 10) and insula after training (all *p* < 0.05). The group × session interaction was significant for BA 25 [*F*(1,38) = 4.652; *p* < 0.05], BA 10 [*F*(1,38) = 10.652; *p* < 0.01], and marginally significant for insula [*F*(1,38) = 3.73; *p* = 0.06], respectively. SPM activation results of Brodmann areas in IBMT group were shown in **Table 1** (after vs. before).

**Table 1:** Statistical parametric mapping (SPM) activation results in integrative body-mind training (IBMT) group (after vs. before).

Brodmann area	*p* value
**Left hemisphere**
BA 32	0.043
Insula	0.026
BA 10	0.001
**Right hemisphere**
BA 25	0.005
BA 32	0.021

**FIGURE 1:**
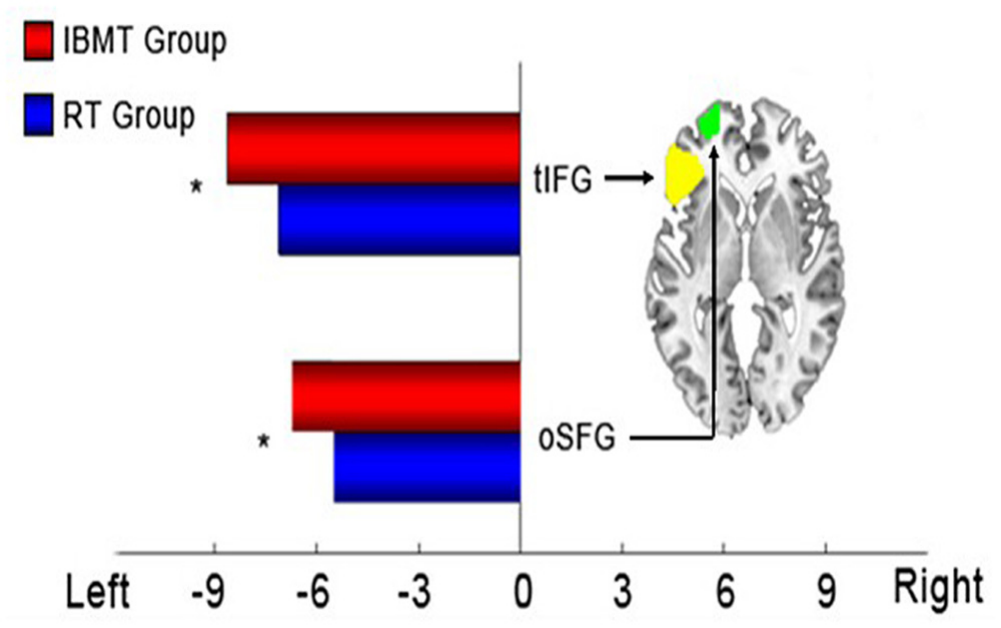
**Statistical results of laterality indexes in frontal regions.** Left: significant frontal laterality differences in integrative body-mind training (IBMT) and relaxation groups. Right: axial section of two frontal regions (*z* = -1). tIFG, triangular part of inferior frontal gyrus; oSFG, orbital part of superior frontal gyrus. *p < 0.05.

There were no significant differences in before vs. after laterality scores in frontal areas between the two groups. Instead, both groups showed significant differences favoring the left hemisphere in tIFG (triangular part of inferior frontal gyrus, *p* = 0.033) and oSFG (orbital part of superior frontal gyrus, *p* = 0.045) as shown in **Figure 1**. The IBMT group revealed larger laterality indexes that implied greater left frontal CBF lateralization, compared to relaxation group after training, but the group × session interaction was not significant.

## DISCUSSION

Consistent with our previous studies, short-term IBMT improves CBF in the midfrontal lobe and insula compared to the relaxation training (Tang et al., 2007, 2009). This result is in line with the neural correlates of mindfulness meditation (Cahn and Polich, 2006; Hölzel et al., 2011; Tang and Posner, 2014). The specific brain areas showing greater CBF following IBMT training suggests that IBMT works, in part, by improving self-regulation (Posner and Rothbart, 2006; Tang et al., 2007; Hölzel et al., 2011; Tang, 2011; Tang and Posner, 2014).

Our study, using the frontal asymmetry analysis, indicated only a few hours of training increases left-sided CBF asymmetry in both groups but IBMT’s increase is greater than that of relaxation training.

In previous studies meditation training promoted positive emotions and degraded negative affect (Kabat-Zinn et al., 1992; Tang et al., 2007; Ding et al., 2014) Studies have found 8-weeks mindfulness meditation produced EEG frontal asymmetry and reduced negative mood state (Davidson et al., 2003). In our previous study of 80 Chinese undergraduates, a few hours of IBMT improved positive moods using a 65*-*item Profile of Mood States (POMS; Tang et al., 2007, 2009; Newberg et al., 2010; Tang, 2011). The current study did not repeat the POMS measures, instead, a brief self-report positive and negative mood scale was used before and after training (Moyer et al., 2011; Ding et al., 2014). After training a significant between-group differences were observed and IBMT group performed better in mood states than relaxation group. However, we did not find a significant difference in the group by time of testing interaction in mood. It is likely that that the brief measure of mood was not as sensitive as the POMS. These findings could also be due to power considerations and sensitivity of imaging techniques (Gatzke-Kopp et al., 2001), but with 20 participants per group our study has a larger number than most imaging studies.

Although studies have shown mindfulness meditation increased left-sided anterior activation, a pattern previously associated with positive affect (Davidson et al., 2003; Moyer et al., 2011) comparing to a wait-list control group, alternation of EEG frontal asymmetry was not detected during transcendental meditation, but the participants’ emotion states were improved (Travis and Arenander, 2004, 2006). Given the low spatial resolution of EEG, it remains to be elusive whether the brain regions involved in these studies are the same. In our study both meditation and relaxation resulted in increased left lateralization, but only IBMT changed mood and brain activity in ACC and insula associated with self-control, consistent with our series of research (Tang et al., 2007, 2009, 2010). Since we did not detect the significant correlation between frontal asymmetry and mood using EEG, future studies will be needed to demonstrate the mechanisms of changes in frontal asymmetry and mood state using ERP/fMRI fusion approach.

Our study shows that few hours of IBMT increases resting CBF in specific brain areas often shown to be involved in attention and self-regulation (Posner and Rothbart, 2006; Tang et al., 2009, 2010; Hölzel et al., 2011; Tang, 2011). However, both IBMT and relaxation training increase frontal asymmetry significantly, although IBMT has a somewhat larger increase. The IBMT group has better overall mood state following training, but it is not clear if the brain differences found in this study are the cause of the changes in affect and mood.

## Conflict of Interest Statement

The authors declare that the research was conducted in the absence of any commercial or financial relationships that could be construed as a potential conflict of interest.

## Footnotes

1 http://www.fil.ion.ucl.ac.uk/spm/

2 http://www.alivelearn.net/xjview8/
